# Successful Insulin Glargine Treatment in Two Pet Guinea Pigs with Suspected Type 1 Diabetes Mellitus

**DOI:** 10.3390/ani11041025

**Published:** 2021-04-05

**Authors:** Theresa Kreilmeier-Berger, Florian K. Zeugswetter, Klaas-Ole Blohm, Ilse Schwendenwein, Elisabeth Baszler, Bernadette Ploderer, Iwan Anton Burgener, Frank Künzel

**Affiliations:** 1 Clinical Department for Small Animals and Horses, Small Animal Clinic, Internal Medicine, University of Veterinary Medicine, 1210 Vienna, Austria; florian.zeugswetter@vetmeduni.ac.at (F.K.Z.); elisabeth.baszler@vetmeduni.ac.at (E.B.); b.ploderer@gmail.com (B.P.); iwan.burgener@vetmeduni.ac.at (I.A.B.); 2 AniCura Tierärztliche Spezialisten, 22043 Hamburg, Germany; klaas-ole.blohm@anicura.de; 3 Clinical Department for Small Animals and Horses, Small Animal Clinic, Ophthalmology Unit, University of Veterinary Medicine, 1210 Vienna, Austria; 4 Tierklinik Rostock, 18059 Rostock, Germany; 5 Department of Pathobiology, Clinical Pathology Platform, University of Veterinary Medicine, 1210 Vienna, Austria; ilse.schwendenwein@vetmeduni.ac.at

**Keywords:** cataract, insulin-dependent, glucometer, phacolytic anterior uveitis

## Abstract

**Simple Summary:**

This is the first case report of two guinea pigs with insulin-dependent diabetes mellitus (DM) successfully treated with long-acting basal insulin glargine. Both animals presented with typical symptoms and laboratory changes like polyphagia, glucosuria and highly elevated blood glucose levels that suggested the presence of a diabetes mellitus. One of the guinea pigs had asymmetric bilateral cataracts. Mostly, a disorder resembling type II-DM in humans seems to be prevalent in guinea pigs. In this case, the animals did not respond to the standard treatment of a dietary change but responded promptly to insulin treatment. The diabetes has remained controlled for over 1.5 years now. Thus far, there is only sparse scientific information on spontaneous type I diabetes mellitus and treatment modalities in pet guinea pigs. We discuss the current literature including up to date diagnosis, treatment, monitoring with the evaluation of different glucometers and long-time follow-up. Moreover, individual ophthalmic abnormalities and management regarding suspected diabetic cataracts are described in detail.

**Abstract:**

Scientific information on spontaneous type I diabetes mellitus (DM) and treatment modalities in guinea pigs is scarce. As most diabetic guinea pigs are overweight and respond to dietary changes, a disorder resembling type II-DM in humans seems to be most prevalent in this species. In the present report, a nine-month-old female intact guinea pig (GP1) was presented because of a cataract and polyphagia. The physical examinations in GP1 and its littermate, GP2, were unremarkable. Laboratory tests revealed hyperglycemia, hyperlipidemia, elevated fructosamine concentrations, and glucosuria in GP1 and GP2. Not responding to dietary changes, an insulin-dependent diabetes mellitus was suspected in both animals. Treatment with 0.5 IU of glargine insulin (Lantus^®^) per guinea pig subcutaneously (s.c.) once daily was initiated in both animals. Monitoring included repeated clinical evaluations and the measurement of plasma glucose and fructosamine concentrations. Capillary glucose concentration was measured using a glucometer, and glucosuria was monitored by dipstick. Blood glucose concentrations decreased quickly in both GPs, and glucosuria resolved. Including several dose adjustments, DM remained controlled for over 1.5 years. Bilateral cataracts and lens-induced uveitis in GP1 were medically managed with only slight progression. This is the first report of guinea pigs with insulin-dependent diabetes mellitus that were successfully treated with long-acting basal insulin glargine.

## 1. Introduction

Scientific data regarding the diagnostics and treatment of spontaneous diabetes mellitus (DM) in pet guinea pigs (GPs) is sparse. In over 40 years, only a few reports about DM in colony bred or pet guinea pigs were published [1,2,3,4,5,6,7,8,9,10,11,12,13,14].

Typical clinical signs in guinea pigs include polydipsia/polyuria, overweight, and diabetic cataract formation [1,2,3,4,6,8,12,15,16]. Secondary bacterial cystitis and idiopathic glomerulopathy, possibly leading to chronic renal failure, are potential sequela of undetected and untreated DM [4,7,10,13,17,18]. Moreover, reproductive dysfunction has been reported in female guinea pigs [2,4,8,9,12,13]. Diagnosis in pet guinea pigs is based on typical clinical signs in combination with glucosuria and hyperglycemia [2,3,4,6,7,8,16]. Additionally, hypertriglyceridemia, but not ketonemia and ketonuria, have been observed [3,4,12,15,19,20].

Insulin-independent DM (type II) is currently the most common diagnosis in diabetic GPs, and treatment in these patients is restricted to food adjustments that often lead to the remission of clinical signs [2,3,4]. Moreover, increased plasma glucose concentrations have been described in diabetic GPs without any response to exogenous insulin, thus indicating strong insulin resistance in at least some of these animals and supporting an insulin-independent DM [9].

Reports of GPs treated with insulin have been restricted to a few case series or single case reports [1,2,3,5,6,7,8]. Despite the use of different insulins, most of these GPs responded to treatment, and clinical signs like polydipsia and glucosuria resolved [1,6,7]. However, information on long-term monitoring and prognosis is lacking. Pathohistological alterations in spontaneous diabetic guinea pigs revealed the severe degeneration of Langerhans islet cells of the pancreas without infiltration by mononuclear cells similar to those in human insulin-dependent DM [8,21]. The pancreatic changes comprised of the severe cytoplasmatic degranulation and hyperplasia of beta-cells combined with the fatty degeneration of the acini as possible signs of the exhaustion of beta cells [13,14]. Additionally, pancreatic fibrosis, hepatic fatty degeneration, glomerulosclerosis, and cataract formation were described [10].

In addition to type I and type II DM, other types of DM like gestagen- and steroid-induced DM have been reported in GPs. Therefore, individual treatment approaches are necessary to optimize treatment success in this species [22,23,24].

Here, we describe two litter mates with diabetes mellitus that did not respond to dietary adjustments and were successfully treated with glargine insulin over 1.5 years following diagnosis.

## 2. Case Presentation

**Figure 1:**
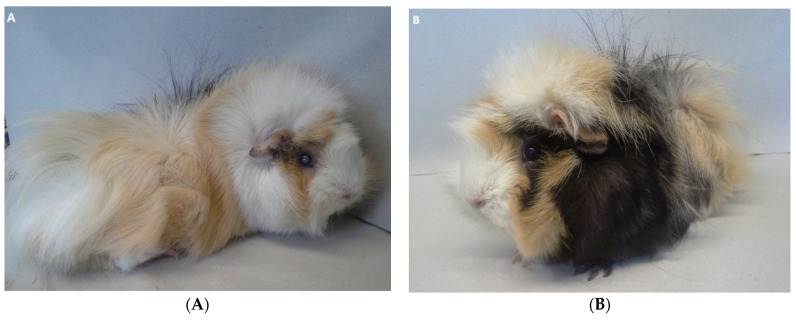
Guinea pig 1 (GP1) (**A**) and guinea pig 2 (GP2) (**B**) at the time of presentation. (**A**) Note cataract formation in the right eye of GP1. (**B**) Physical examination was unremarkable in GP2. Both guinea pigs showed an age-appropriate body condition and were not overweight.

A nine-month-old female intact guinea pig (GP1) and its female littermate (GP2) were referred to the Clinic for Small Animals of the University of Veterinary Medicine Vienna for the further diagnostic work-up of a rapidly developing lenticular opacity in the right eye of GP1. GP2 was presented for a routine clinical examination. While unilateral lens opacity and polyphagia were reported in GP1 by the owner, GP2 was considered healthy. Their food consisted of hay, salad, cucumber, carrots, apples, and grains in both animals. General physical examination confirmed cataract formation in the right eye in GP1 but was unremarkable in GP2 (Figure 1A,B). Neither guinea pig was overweight (body condition score: 5/10; ideal weight), with body weights of 950 g (GP1) and 920 g (GP2).

**Figure 2:**
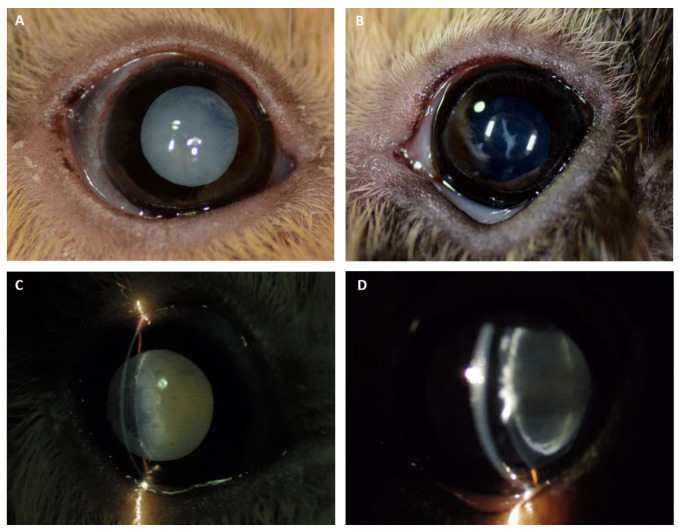
Cataract formation in GP1. (**A**) The right eye of GP1 showed a complete cataract with water clefts along the anterior suture lines. (**B**) The left eye revealed a spike-like immature anterior cortical cataract. (**C**, **D**) Slit-lamp photographs depicting a shallower anterior chamber (intumescent cataract) and mild cortical liquefaction along the suture lines in the right eye, and the perinuclear anterior cortical cataract localization in the left eye.

An ophthalmic examination in GP1 revealed a mature cataract with water clefts along the anterior suture lines in the right eye and a left-sided immature cataract (Figure 2A,B). Slit-lamp biomicroscopy yielded a shallowed anterior chamber of the right eye that was consistent with an osmotic lens fiber swelling and subsequent intumescent cataract, whereas in the left eye, the cataract was localized to the anterior lens cortex (Figure 2C,D). A moderate and mild phacolytic anterior uveitis was apparent in the right and left eyes, respectively, accompanied by a darkened iris color considered to be a consequence of chronic intraocular inflammation. GP2 showed no ophthalmic abnormalities.

**Figure 3:**
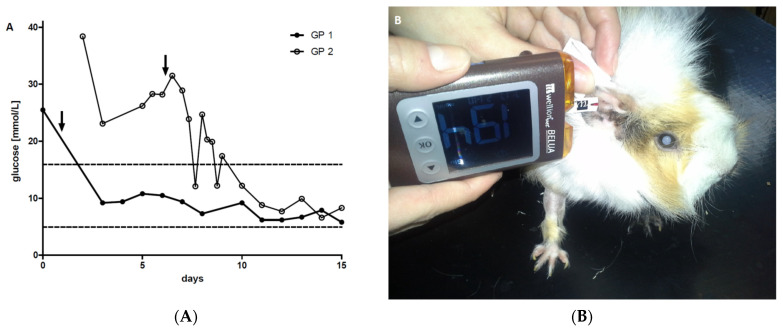
Blood glucose concentrations determined by a glucometer at the beginning of insulin treatment. (**A**) After the initiation of insulin therapy, the glucose concentrations of the two guinea pigs decreased rapidly (within 1–2 days). Dashed lines indicate upper and lower reference limits [26]. Arrow (↓) depicts the initiation of insulin treatment. Full and open symbols resemble GP1 and 2, respectively. (**B**) Representative picture of blood glucose measurement by glucometer (WellionVet Belua^®^, MED TRUST GmbH, Marz, Austria) in GP1.

**Figure 4:**
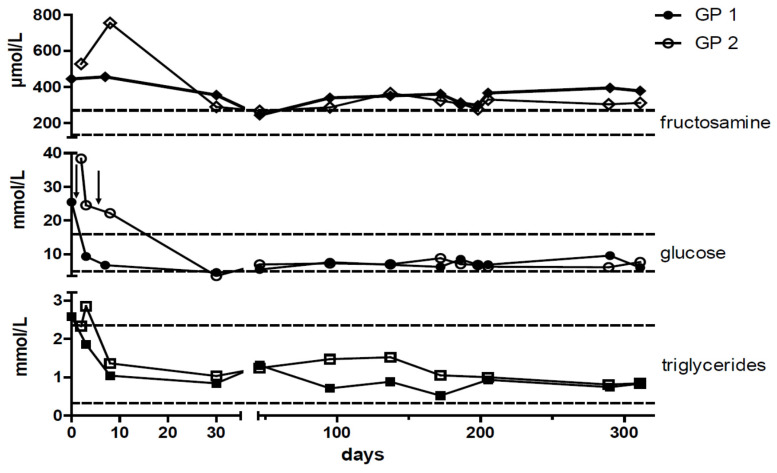
Timeline of plasma fructosamine, glucose, and triglyceride concentrations. After the initiation of insulin therapy blood glucose, fructosamine, and triglyceride concentrations decreased. All three parameters were simultaneously measured at the individual time points. Arrow (↓) depicts the initiation of insulin treatment. Dashed lines indicate upper and lower reference limits [26]. Full and open symbols resemble GP1 and GP2, respectively.

Urinalysis using Combur9^®^ test strips (Roche Diagnostics, Rotkreuz, Switzerland) showed severe glucosuria (>55 mmol/L) without ketonuria in both GPs [25]. Other results of urinary dipstick testing with a normal pH of 9, as well as the evaluation of urinary sediment, were unremarkable in both GPs. The blood biochemistry profile consisted of increased plasma glucose concentrations of 25.5 mmol/L in GP1 and 38.4 mmol/L in GP2 (COBAS 6000 c 501, hexokinase enzyme assay; reference range: 4.9–15.9 mmol/L) (Figure 3A and Figure 4; Table 1) [26].

In addition, both guinea pigs had increased plasma fructosamine concentrations of 445 µmol/L in GP1 and 527 µmol/L in GP2 (upper reference limit of local laboratory = 271 µmol/L). Triglyceride concentration was slightly increased in GP1. In GP2 this parameter was close to the upper reference limit of 2.35 mmol/l at presentation and increased marginally above it on the consecutive day (Figure 4 and Table 1). Hematology and other blood biochemistry test results were within normal limits.

Over a period of ten days, feeding was changed to an exclusively high fiber diet consisting of hay, fresh and dried herbs, cucumber, and salad. As dietary changes did not induce improvement, insulin-dependent diabetes mellitus was considered and treatment with glargine insulin was initiated. Both GPs received 0.5 IU of glargine insulin (Lantus^®^, Sanofi-Aventis, Paris, France) per animal subcutaneously (s.c.) once daily using 100-unit syringes (0.3 mL ½ units, BD Micro-Fine Demi, BD Medical, Franklin Lakes, NJ, USA). To avoid insufficient resorption through subcutaneous fat deposits in the back neck of guinea pigs, insulin was subcutaneously administered on the left or right side of the thorax or abdomen. An exclusively high fiber diet including fresh food and hay was nonetheless maintained. Symptomatic topical, preservative-free, diclofenac-sodium treatment (Voltaren Ophtha ABAK^®^; three times daily in the right and two times daily in the left eye) was initiated in GP1. Ophthalmic re-examination confirmed a significant improvement of lens-induced anterior uveitis two weeks later.

For the initial insulin treatment and monitoring, both guinea pigs were hospitalized. Monitoring included clinical examinations and daily measurements of capillary and urinary glucose concentrations. Free catch urine samples were collected by gentle manual pressure on the bladder, and urine glucose concentrations were determined using urine dipsticks (Combur9^®^, Roche Diagnostics, Rotkreuz, Switzerland). Whole blood glucose concentrations using the ear vein prick technique on the pinnae were determined with a veterinary glucometer approved for cats, dogs, and cows (WellionVet Belua^®^, MED TRUST GmbH, Marz, Austria) (Figure 3B). For each species, a specific chip had to be applied to the glucometer before measurement. To validate to the usage of the glucometer and to determine the most appropriate chip for use in guinea pigs, blood glucose concentrations determined by glucometer were compared to the plasma glucose concentrations determined by the laboratory method at the university stated below. The glucometer WellionVet Belua^®^ (MED TRUST GmbH, Marz, Austria) with the chip for dogs showed the best agreement with plasma glucose concentrations (n = 13) that were simultaneously determined by the hexokinase enzyme assay (COBAS 6000 c 501) (bias = 0.33; 95% confidence interval (CI): from −4.17 to 4.84; Bland–Altman plot) compared to the chips for cats or cows (bias = −1.32; 95% CI: from −7.16 to 4.54; and bias = −2.68; 95% CI: from −7.96 to 2.6; Bland–Altman plot, respectively) in four other guinea pigs with diabetes mellitus with glucose concentrations between 4.4 and 25.5 mmol/L. The reproducibility of the glucometer measurements was good, with a median standard deviation of 0.6 mmol/L within day precision.

After discharge from the hospital, the guinea pigs underwent re-checks at monthly intervals for the first 6 months and then every 3 months throughout the observation period (Figure 4). Blood glucose and triglyceride concentrations returned to normal, and glucosuria resolved completely within a few days (Figure 3A and Figure 4). The diabetes remained clinically controlled in both animals. The insulin dosage was adapted to correspond to blood glucose concentrations and varied between 0.5 and 0.75 IU per GP once daily in GP1 and between 0.5 IU per GP once daily to every other day in GP2. Insulin dosage was reduced in GP2 due to blood glucose concentrations around the lower reference range to avoid hypoglycemic episodes (Figure 4). Overall plasma glucose concentrations remained below the upper reference limit and ranged from 4.6 to 10.8 mmol/L (mean = 7.8 mmol/L; SD = 1.3 mmol/L) in GP1 and from 3.5 to 12.2 mmol/L (mean = 8.0 mmol/L; SD = 1.2 mmol/L) in GP2 (Figure 4 and Table 2). Fructosamine concentrations decreased more than 100 µmol/L in both guinea pigs but remained slightly above the reference range despite plasma glucose concentrations having dropped to the lower reference range (Figure 4). Glucosuria resolved within a few days after the initiation of treatment and remained absent throughout the entire observation period. Concordantly, the quantitative determination of urine glucose concentrations measured by the urine glucose/creatinine-ratio (UGCR) remained below the detection limit during the entire observation period. Neither urine ketones (acetoacetate and acetone) nor plasma β-hydroxybutyrate were detectable during the observation period. Both animals showed a normal age-related increase in weight of up to 1000 g (GP 1) and 1015 g (GP 2).

Ocular signs were monitored after two weeks and six and twelve months in both guinea pigs. Due to controlled phacolytic anterior uveitis, diclofenac-sodium eye drops in GP1 were tapered to effect. No active inflammation was clinically recognizable with a twotimes daily (right eye) and once daily (left eye) topical maintenance treatment (Voltaren Ophtha ABAK^®^). A mild punctate anterior cortical cataract extension developed over one year in the left eye adjacent to the initial spike-like opacities. The overall stage of cataract maturity in GP1 nevertheless did not change compared to the initial presentation and remained stable without evidence of lens capsule rupture in the right eye. As both eyes appeared comfortable and GP1 exhibited no obvious visual impairment, cataract surgery was not reinforced. Ophthalmic findings in GP2 remained within normal limits throughout the follow-up period.

Insulin injections were well-tolerated by the guinea pigs and could be adequately administered by the owner. No signs of inflammation, infection, or other skin lesions occurred at the injection sites.

Unfortunately, GP2 developed a skin necrosis at the distal part of the front leg following blood sampling and using a bandage to stop bleeding. Skin necrosis had to be surgically treated applying a skin flap. Both animals are well and still under treatment after 1.5 years since first presentation.

## 3. Discussion

Spontaneous DM is a rather well-described endocrine disorder in GPs. Despite this, most information is based on anecdotal evidence instead of scientific data. Furthermore, etiology is still under discussion. DM in humans and other species like dogs and cats is caused by various underlying pathologies that lead to defects in insulin secretion and/or insulin sensitivity in target tissues, suggesting that a similar approach is necessary in GPs [2,27].

The two GPs of the present study resembled an insulin-dependent/type 1 DM in humans because they were young, of normal weight, and saw the resolution of their hyperglycemia and glucosuria after insulin administration but not after dietary changes. Nevertheless, it cannot be ruled out that diet adjustment contributed to the treatment response. No signs of spontaneous remission were observed until the end of the study and the GPs continued to be dependent on insulin treatment, although the withdrawal of insulin was never attempted. An advanced stage of insulin-dependent type II seemed less likely given the age and normal body weight of the two GPs.

According to the literature, a diet-responsive DM (type II) seems to occur most frequently in pet guinea pigs, thus leading to spontaneous remission after food adjustment and weight reduction [2,4]. Additionally, the oral antidiabetics currently used in human insulin-resistant type II DM have had varying success [2,6,7,18,27,28]. On the other hand, the previously found pathological alterations resembled more a human juvenile (type I) DM with an onset at an early age [13,14].

A gestagen-induced diabetes mellitus, likely due to hormonally active ovarian cysts, was described recently [22]. However, no signs of ovarian cysts were existent in the GPs of the present study, and they most likely would not have responded adequately to initial insulin therapy [16,22].

As neither GP received medications including glucocorticoids, steroid-induced diabetes mellitus (as previously described in experimental settings) could be ruled out [23,24]. Simultaneously, a spontaneous hyperadrenocorticism in two young GPs was considered unlikely and no other signs such as alopecia, potbelly, or muscle wasting were observed [29].

A viral or contagious cause of diabetes mellitus in guinea pigs was discussed about 40 years ago with the identification of virus-like particles in the urine of diabetic GPs in an isolated breeding colony [11,12,13]. Since then, no further investigations have been conducted. A hereditary background of diabetes mellitus has also been suspected, as it was prevalently observed in selected strains or colonies of guinea pigs [8,10].

Apart from polyphagia and cataracts in GP1, no clinical signs of DM were detectable in the animals of the present study. This was interesting, as the key signs polyuria/polydipsia (PU/PD) were not reported by the owners.

Occasionally, bacterial cystitis is described in diabetic guinea pigs as a result of glucosuria and altered immune response [4,17]. Though severe glucosuria was detectable, urinalysis and clinical examination did not indicate cystitis in GP1 or GP2.

Cataracts are commonly reported in guinea pigs with DM [8,10]. In a survey investigating ocular diseases in 1000 guinea pigs, 17.4% had cataracts. Thirty-four of these animals, with the majority (n = 27) being diabetic, had bilateral mature cataracts [30]. Unilateral cataracts were described in a diabetic rabbit and a chinchilla with elevated blood glucose concentrations [1]. GP1 constitutes the first description of an asymmetric bilateral cataract with an assumed diabetic cause in this species. Due to the pathophysiology of diabetic cataracts, the asymmetric hypertonicity of the lenses in one individual with generalized elevated plasma and aqueous humor glucose concentrations appeared odd. In humans, unilateral and reversible diabetic cataracts were speculated to be associated with asymmetric subcapsular changes, and, accordingly, different damage of the lens fibers may be responsible for variable glucose permeability of the lenses [31]. As in young human patients, this finding might also indicate an early sign of DM in GPs [32]. The less pronounced lenticular changes in the contralateral eye of GP1 were easily missed without a detailed slit-lamp examination.

Higher glucose concentrations and correspondingly higher sorbitol accumulations within the lens capsule could have triggered cataract development in GP1, whereas more constant and, on average, lower glucose concentrations might explain the reduced risk of cataract formation in GP2. Additionally, individual differences in the hexokinase saturation or in the amount and activity of aldose reductase between the GPs or between both eyes were possible. High amounts of aldose reductase in the lens of GPs imply significant a contribution to the pathogenesis of diabetic cataract formation in this species [33]. The lens epithelial expression of silent information regulator protein-1 and p53 was significantly increased in early diabetic cataracts in rats and might merit further investigation in GPs [34]. The observed intumescent lens with water clefts is characteristic for a rapidly progressed cataract, which makes a congenital, inherited, or chronic dietary cause less likely.

The diagnosis of DM was assumed by increased plasma glucose and fructosamine concentrations, as well as glucosuria. The determination of urine glucose concentrations by urine dipstick has especially been shown to be a non-invasive and particularly useful initial indicator in the diagnostic work-up of GPs with suspected DM [1,2,3]. However, the determination of plasma glucose and fructosamine concentrations is recommended for the confirmation of diagnosis of DM in GPs to avoid misdiagnosis and unnecessary treatment. Ketones could not be detected in the blood (β-hydroxybutyrate) or the urine (acetoacetate and acetone) in both guinea pigs. As the assays used in the current case report have not been validated for the use in GPs, data have to be cautiously interpreted, but results were in line with earlier studies where DM was not associated with ketosis in GPs [12,35]. Noteworthy, ketosis in GPs is commonly observed due to anorexia and might be found in female guinea pigs during pregnancy as well [35,36,37].

Plasma triglyceride concentrations were increased in both of the GPs, thus resembling the results of other studies [4,15]. Hyperlipidemia is common in all types of diabetes and can be explained by the omitted effects of insulin on lipid metabolism, especially the hormone-sensitive lipase [27]. As expected in case of treatment success, triglyceride concentrations returned into the reference range after the initiation of insulin treatment in both GPs of the present study.

Suspected insulin-dependent DM, as well as the rapidly developed asymmetric bilateral cataracts in GP1, led to the decision for insulin treatment. This therapeutic approach for diabetic GPs has so far only vaguely been described in case reports [1,5,6,7,8]. Like the GPs of the present study, one case report documented insulin treatment in two diabetic littermates, with bilateral immature cataracts in one of them. Both were treated with a no-longer-available combined bovine/porcine lente insulin (1 IU/kg s.c. once daily), and although glucose decreased, concentrations stayed above the stated upper reference limit [1]. Other published insulin preparations used with success in GPs with spontaneous DM include porcine and human NPH (neutral protamine Hagedorn) insulins with dosages ranging from 1 to 3 IU/GP twice daily and a porcine lente insulin (Caninsulin^®^) validated for the use in dogs and cats [5,6,7]. All of these preparations led to the clinical improvement and resolution of glucosuria in the treated GPs. However, lente insulin was reported to have a short duration of action. In contrast, no responses to treatment were observed in a colony of diabetic GPs treated with a bovine insulin preparation [8]. Possible explanations include the non-effectiveness of bovine insulin, inadequate dosage, or a severe insulin-resistant state in GPs.

As GPs eat constantly throughout the day (which does not lead to postprandial glucose peaks) and the owners argued that they would not be able to administer insulin injections twice a day, a long-acting insulin (glargine) was used in the current study. The smallest possible starting dose of 0.5 IU per GP once daily using syringes for children (BD Micro-Fine Demi, BD Medical, Franklin Lakes, NJ, USA) was applied. Though the fine dosing of insulin in 0.1 IU steps is possible using an electronic insulin pen (Pendiq^®^, Moers, Germany), this pen is costly and does not allow for dosages below 0.5 IU. As GP insulin has only 1–5% the activity of other insulins, 0.5 IU per GP (once daily) was considered a rather high dose and was used with concern [38].

Insulin application showed an immediate effect that was represented by the normalization of plasma glucose concentrations within one-to-two days in both GPs. Only slight adjustments of the dosage had to be made throughout the course of treatment.

Though the use of fructosamine as a monitoring tool is a matter of controversial debate in cats and dogs and has rarely been used in GPs, plasma fructosamine was used as an additional tool in the present study [26,39,40]. Noticeably, plasma fructosamine concentrations decreased within only a few weeks after the start of the insulin treatment but stayed slightly above the upper reference limit during the entire observation period. This might indicate hyperglycemic periods that contributed to slight cataract progression in the left eye of GP1, or, as in dogs, increased fructosamine concentrations are possible even in well-controlled diabetic GPs, thus indicating individual differences of protein glycation [39]. Further studies are encouraged.

Phacolytic anterior uveitis was bilaterally controlled throughout the entire observation period by preservative-free diclofenac-sodium eye drops. Neither ocular nor systemic side effects were noticed with this long-term maintenance treatment. Furthermore, the data and ophthalmic monitoring in the present study suggested that systemic insulin therapy was beneficial in preventing significant cataract progression. The welfare of GP1 was not noticeably compromised due to its cataracts and without phacoemulsification.

With the normalization of blood glucose concentrations due to insulin therapy, glucosuria also quickly resolved. In accordance with other studies, urinary dipsticks proved very useful as a basic tool for the monitoring of insulin-therapy because urine can be easily and non-invasively sampled, even by the owner [2,7]. However, hypoglycemic episodes remain undetected.

The most common side effect of insulin administration is hypoglycemia [27,39]. Thus, blood glucose concentrations were frequently determined by a glucometer to detect possible hypoglycemic events. As no glucometer has yet been validated for use in guinea pigs, we evaluated a glucometer designed for dogs, cats, and cattle. The chips for dogs with only a slight positive bias correlated best with the plasma glucose concentrations determined by a conventional method (gold standard). Hence, these were chosen in the present case report. Neither clinical signs nor plasma glucose concentrations indicating hypoglycemia were observed during the entire observation period. The lack of hypoglycemia may support the need for insulin therapy. No signs of spontaneous remission after insulin therapy occurred, thus underlining the plausibility of an insulin-dependent DM [2,4,16].

The rapid decrease of blood glucose concentrations to the lower reference limit, the enormous decrease of serum fructosamine concentrations and the disappearance of glucosuria, as well as only minimal cataract progression in GP1, strongly indicated the sufficient control of the diabetes mellitus in these two GPs.

No side effects were observed during insulin treatment in the present case report. However, skin necrosis developed in GP2 soon after a venous puncture. A bandage, used to stop bleeding at the puncture site but concurrently preventing normal blood circulation, was assumed to be the most likely cause of skin necrosis, as previously already observed in a diabetic GP (personal observation). This suggested that GP with DM are particularly sensitive to pressure bandages and therefore must be avoided after blood sampling.

## 4. Limitations

This case report only included two patients in a clinical setting. As the GPs are still alive, no pathohistological examinations, e.g., of the Langerhans islet cells and lens fibers, could be performed and the cause of DM could not be verified. The main drawback of this report was the small sample size and the therapeutic intent that prohibited the withdrawal of the drug for a proof of concept. Therefore, it must be kept in mind that there is no statistical power in this report, and it is only descriptive. Moreover, animals may have individual factors. In addition, males and females can have different levels of responsiveness to certain drugs. Further studies are warranted.

## 5. Conclusions

In conclusion, insulin-dependent DM exists in pet GPs, and treatment with insulin glargine is an efficient, safe, and practicable option. Moreover, insulin therapy appears to be beneficial to prevent significant diabetic cataract progression. The determination of urinary glucose by a dipstick, as well as the determination of blood glucose concentrations using a glucometer, can be recommended for the monitoring of insulin therapy. This study also showed that the ear vein prick technique is a valuable monitoring tool in GPs and that the determination of blood fructosamine concentrations may be an appropriate tool for the long-term assessment of insulin treatment in guinea pigs. This publication should serve as a starting point for more structured experimental studies. Further studies with a larger cohort of GPs and the investigation of risk and protective factors contributing to naturally occurring diabetic cataracts in clinical cases are warranted.

## Figures and Tables

**Table 1:** Clinical biochemistry parameters of both guinea pigs (GPs) at the time of initial diagnosis.

Parameter	GP 1	GP 2	Reference Range ^1^
Glucose	25.5 ↑	38.4 ↑	4.9–15.9 mmol/L
Fructosamine	445 ↑	527 ↑	134–271 µmol/L
Beta-hydroxybutyrate	0.05	0.07	<1 mmol/L
Creatinine	35.4	30.5	34–166 µmol/L
Urea	6.2	6.2	3.3–10.3 mmol/L
Total protein	65.4	66.2	48.9–73.9 g/L
AP	87	161	<418 U/L
ALT	23	21	0–61 U/L
GLDH	4.71	2.41	<17 U/L
Triglycerides	2.6 ↑	2.3	0.33–2.35 mmol/l

^1^ Reference ranges were used as previously published [25]. ↑ = elevated; AP = alkaline phosphatase; ALT = alanine aminotransferase; GLDH = glutamate dehydrogenase.

**Table 2:** Glucose concentrations (mmol/L) by glucometer (WellionVet Belua^®^) and by gold standard method (COBAS hexokinase assay) after the initiation of insulin treatment *.

	n	Mean	Range	SD	
Glucometer	54	8.0	6.3–10.8	1.2	GP1
Gold standard	12	7.0	4.6–9.5	1.5	
All data	66	7.8	6.3–10.8	1.3	
Glucometer	53	8.0	5.3–12.2	1.3	GP2
Gold standard	11	8.1	3.5–22.1	1.4	
All data	64	8.0	3.5–22.1	1.2	

Glucose reference range: 4.9–15.9 mmol/L; * day 3 in GP1 and day 4 in GP2; n = number of blood samples; SD = standard deviation.

## Data Availability

The data supporting the results reported in the article can be found in the animal hospital information system (TIS^®^).
